# On Criminal Lunacy
*Suggestions for the Future Provision of Criminal Lunatics. By W. Charles Hood, M.D., Resident Superintendent Physician of Bethlehem Hospital. London: Churchill. One vol. 8vo, 1854.


**Published:** 1854-07-01

**Authors:** 


### ART. V.—ON CRIMINAL LUNACY.*

WITHIN the last few years the public, professional, and legislative attention has been particularly directed to the question of criminal lunacy. In connexion with this subject, several valuable pamphlets have lately been published. We refer specially to the interesting *brochures*

* Suggestions for the Future Provision of Criminal Lunatics. By W. Charles Hood, M.D., Resident Superintendent Physician of Bethlehem Hospital. London: Churchill. One vol. 8vo, 1854.

of Drs. Wood and Bucknill, reviewed in previous numbers of this journal. The most recent work on the subject is that now under our critical consideration. Dr. Hood, from the great attention he has paid to this matter, and from the position he occupies as the resident physician of one of the largest and most important public asylums in this country, is admirably qualified to offer suggestions in reference to the future treatment of criminal lunatics.

Dr. Hood's work displays great research, and considerable literary ability. We propose placing before our readers a short analysis of his labours. We regret that our space will not admit of our quoting more at length from this valuable publication. When speaking of the warrants for criminal lunatics, Dr. Hood observes :—

"Here it may be well to explain that insane criminals are sent to lunatic asylums under two descriptions of warrant,—viz., the royal warrant, executed by command of the reigning sovereign, and a warrant signed by one of the principal Secretaries of State. If a jury acquit a person charged with treason on account of insanity, or a person indicted for any offence be found, upon arraignment, insane, the Act 39 & 40 Geo. III., c. 94, provides that 'the court before whom such trial shall be had shall order such person in strict custody, in such place and in such manner as to the court shall seem fit, until his Majesty's pleasure shall be known; and it shall, therefore, be lawful for his Majesty to give such order for the safe custody of such person, during his pleasure, in such place and in such manner as to his Majesty shall seem fit.' This Act appears to have been suggested by a difficulty which occurred upon the trial of Hadfield, who remained for many years an inmate in Bethlehem Hospital, and there died in the year 1849. After the very memorable speech which was delivered on that occasion by Lord Erskine—a speech unrivalled for the beauty of its language, the dignity of its style, and the perspicuity of its argument; the insanity of the unfortunate man was so clearly demonstrated, that Lord Kenyon, presiding as Chief Justice, stopped the case, and directed the jury to return a verdict of 'not guilty;' but then arose the question, how the prisoner was to be disposed of? 'For his own sake,' said Lord Kenyon, 'and for the sake of society at large, he must not be discharged, for this is a case which concerns every man of every station, from the King upon the throne to the beggar at the gate—people of both sexes and all ages may, in an unfortunate frantic hour, fall a sacrifice to this man, who is not under the guidance of sound reason, and, therefore, it is absolutely necessary, for the safety of society, that he should be properly disposed of, all mercy and humanity being shown to the unfortunate creature; but, for the sake of the community, he must somehow or other be taken care of, with all the attention and all the relief that can be afforded him.' Hereupon, the counsel for the Crown, and the counsel for the defendant, agreed that the safety of the community required that he should be taken care of. 'It is laid down in some books,' said the former (Sir John Mitford, afterwards Lord Redesdale),

'that by the common law, the judges of every court are competent to direct the confinement of a person under such circumstances.'—'That may be, Mr. Attorney-General,' interposed Lord Kenyon, 'but, at present, we can only remand him to the confinement he came from; but means will be used to confine him otherwise in a manner much better adapted to his situation.' It was then suggested by Mr. Garrow (afterwards a Baron of the Exchequer), that 'it would be for the benefit of posterity if the jury would state in their verdict the grounds upon which they gave it,—viz., that they acquit the prisoner of this charge, he appearing to them to have been under the influence of insanity at the time the act was committed. There would then,' he added, 'be a legal and sufficient reason for his confinement.' This recommendation was adopted by the jury, who returned a verdict in these terms. Thus originated the form of verdict now commonly returned in cases of this description."

Dr. Hood then directs the attention of his readers to the cases of Margaret Nicholson, and Frith,—cases that led to some important modifications of the criminal law. He says, that in

"August 8th, 1786, after Margaret Nicholson had attempted to assassinate George III. with a knife, having approached his Majesty's person under the pretext of delivering a petition, she was taken into custody, and afterwards examined at Whitehall by the Lords of the Privy Council. Upon the evidence of Dr. John and Dr. Thomas Monro, she was found insane. It was proposed to commit her to Tothill-fields prison, but this was objected to, upon the ground that she was a State prisoner; 'in consequence, therefore, of the determination' of the Privy Council, it is stated that 'the unhappy woman was conveyed to a cell prepared for her in Bethlehem.'

"In the January of 1790, another lunatic, named John Frith, attempted to assault his Majesty by throwing a stone at the royal carriage, as his Majesty was going in state to the House of Peers. This man, who was obviously insane, did not meet with so much clemency as Margaret Nicholson, for after undergoing several examinations at the Treasury Office, Whitehall, by the Attorney-General, in the presence of the principal Ministers of State, he was committed to Newgate, and there imprisoned nearly two years. It was not until the 11th of December, 1791, that he was put to the bar, charged with committing high treason by throwing a stone at his Majesty. The affidavits of a physician and surgeon were produced, stating that they had attended the prisoner since his confinement, and had examined into the state of his mind, and found him insane. The Attorney-General said he had seen the affidavits, and was convinced of the truth of them: and had authority to inform the court that he was in possession of the King's sign manual, by which his Majesty consented to the prisoner being discharged from the gaol of Newgate, upon condition that security was given that he should be confined in some proper place as a lunatic, or in some other manner taken care of, so as to answer his Majesty's most gracious intentions. Bail was accordingly produced, and the prisoner ordered to be liberated.

"These cases, and the subsequent one of Hadfield, showed clearly the necessity for some further legislation on the subject, and accordingly, the trial of Hadfield having taken place on the 26th June, 1800, a few days afterwards, on the 30th June, the Attorney-General, in the House of Commons, moved for leave to bring in 'The Treason Bill, and the Insane Offenders' Bill.' The object of the 'Treason Bill' was to give the life of the Sovereign the same protection as the law afforded to the meanest subject of the realm; for previous to the passing of this statute, it was necessary, on an indictment for high treason, even where the life of the Sovereign had been openly attempted, to prove the overt act by two witnesses; and a number of forms were provided by the old law, which were salutary and proper in cases where the charge wore a political aspect; where the treason consisted in an alleged rebellious conspiracy, or might be considered in the nature of constructive treason; or where the prosecutor might have an interest in bringing home the charge to the person accused: but where the life of the Sovereign was attempted by a simple act perpetrated in the face of the public, the facilities for conducting the prosecution were embarrassed, and the trial could not be conducted in the same way as if the party indicted were a common subject. The 'Treason Bill,' therefore, provided that, in cases where a person was indicted for the assassination of the King, or any direct attempt against his life, the offender shall be tried in the same manner as if charged with murder; but be punishable, if convicted, by execution as in other cases of high treason."

Chapter II. of Dr. Hood's volume is devoted to the investigation of the "statistics of insanity," and Chapter III. to the consideration of the "statistics of criminal lunacy." The question of general statistics, discussed in the former chapter, is deserving of much attention. Dr. Hood clearly demonstrates the want of precise and accurate statistical data, in reference to insanity, and points out with great force the discrepancy of opinions existing among those who have attempted to elucidate this intricate subject. Dr. Hood, as the result of his investigation, deduces the following inferences:—

"*First*—That insanity is a disease which, making every allowance for the increase of the population, has been greatly on the increase.

"*Second*—That insanity is more prevalent in some counties than it is in other counties, and predominates more in agricultural districts than in towns.

"*Third*—That insanity, in proportion to the population of the two classes, prevails to a greater extent among paupers than among persons belonging to the middle and upper classes of society.

"*Fourth*—That the number of females afflicted with insanity, making allowance for the excess of the female population, is greater than the number of males."

The chapter on the "statistics of criminal lunacy" is replete with valuable tabular matter. The following are the author's results:—

| | |
|---|---|
| "Proportion of the Insane to the number of Commitments . | 1 to 920 |
| Proportion of the Insane to the number of Convictions . . | 1 to 684 |
| Annual average of Offenders found Insane . . . . . . . | 29 |

"Hence it appears, that during fifteen years, from 1838 to 1852 inclusive, there were 408,617 offenders committed for trial, of whom 301,977 were convicted, 106,199 acquitted, and 441 detained as insane, 204 having been found insane on arraignment, and 237 having been acquitted on the plea of insanity. Furthermore, we may observe by these tables, that the annual average number of commitments in each of the fifty-two counties of England and Wales was, during this period, 7858, and the annual average number of offenders found insane on arraignment, or acquitted on the plea of insanity, in each county, was 8·25.

"The greatest number of criminal lunatics, during a period of fifteen years, belonged to the second class of offenders—those charged with committing offences against property, without violence; 156 having been accused of larceny, of whom 87 were found insane upon arraignment, and 69 acquitted upon the plea of insanity. It has been generally assumed that the majority of criminal lunatics commit the higher offences included in the first class,—those against the person; murder, attempts to murder, manslaughter, &c.; but this appears to be an error. Of the above, 108 were accused of murder, of whom 33 were found insane upon arraignment, and 75 acquitted upon the plea of insanity; 32 were indicted for attempts to murder, of whom 15 were found insane upon arraignment, and 17 acquitted on the plea of insanity. Furthermore, 27 were indicted for stabbing and wounding with intent to maim and disfigure, of whom 13 were found insane on arraignment, and 14 acquitted as being insane. Lastly, 10 were committed for manslaughter, of whom 5 were found insane upon arraignment, and 5 acquitted upon the plea of insanity."

Among the numerous suggestions that have been made for the future treatment of criminal lunatics, it has been proposed that one great central or state asylum should be erected for their common reception. The author, however, entertains serious objections to this proposal. He thinks great evils would result from congregating all the criminal lunatics of the country under one roof. He asks, whether it would be fair or humane to incarcerate a lady or a gentleman who may, under a momentary insane impulse, have committed a trivial misdemeanour, in the same ward, or even in the same establishment, with women or men belonging to the lowest classes of society, who may have committed revolting and nameless offences? We must confess that we do not see how this obvious objection can be answered. We do not conceive it to be practicable in the present state of society to have two distinct classes of state asylums for criminal lunatics,—one being for the reception of criminal ladies and gentlemen, and the other persons connected with the middle and lower classes of society. Dr. Hood justly remarks:—

"There is no family in the kingdom—from the domestic circle of the highest peer of the realm down to that of the humblest peasant—that may not be stricken with the calamity of insanity; and a very trivial transgression may render the afflicted person amenable to trial in a public court of justice. Acquitted as being insane, is the hapless offender—who may be highly connected, well-educated, and habitually sensitive and refined,—to be cast among coarse and ruthless ruffians, whose hands—insane as they may have been when they committed such offences—have been tainted with the most atrocious and loathsome crimes? The object of the legislature is to restrain, not to punish, these unfortunate beings."

Would not the establishment of a state lunatic asylum for the centralization of all criminal lunatics materially interfere with the great and humane object of the Legislature, as propounded in the Act of Geo. II., c. 5, s. 17, which affirms that "they (criminal lunatics) shall be kept, maintained, and CURED?"

If lunatics were only permitted to associate with each other, would not the chances of their recovery be materially diminished, and the designs of the Legislature be frustrated? Dr. Hood raises other objections to the proposal. He says, very justly—

"In whatever county the proposed central asylum be erected, the relations and friends of the afflicted, living in remote places, would have to travel a great distance to visit them;—an inconvenience which would press heavily on the poorer classes, who would probably have to perform the journey on foot, losing many days' work in the performance of a filial, or it may be, a parental duty. This grievance, which would be undoubtedly incurred, merits consideration. Again: the concentration of evil-doers, insane or sane—more especially those afflicted with any disease, bodily or mental—is notoriously impolitic; and it may fairly be predicted that such an asylum would soon assume the character of a prison rather than that of a curative hospital. It would be regarded in the light of a bastile, and would be, however well conducted, desecrated by no slight amount of popular odium. Already a great prejudice exists in the public mind against all lunatic asylums; the result of which is, that many families are reluctant to place their immediate relations under proper medical treatment in the early and curative stages of the malady. To this cause may be ascribed, in some measure, the increase of insanity; for were other diseases in their incipient stages equally neglected, there can be no doubt that the bills of mortality, in respect to them, would be very greatly augmented."

Our author, considering that sufficient attention has not been paid to the classification of criminal lunatics, suggests, that those who have committed the higher class of offences—murder, treason, sedition, &c., who are prosecuted and supported as State prisoners at the expense of the Government, should, under a Queen's warrant, be confined in Bethlehem Hospital; but that insane persons, guilty of minor offences, might, under a warrant of the Secretary of State, be sent to their respective county asylums,—the parishes to which they belong being liable for the expense of their maintenance:—

"Were these suggestions adopted, there would be no fear of the accommodation for criminal lunatics becoming exhausted; and the burden of supporting them—dispersed over different counties—would be fairly equalised. The resident superintendents would soon become familiar with the history of every case, the peculiar features of the malady, and the temper and disposition of such persons; and, taking every circumstance into consideration, would be best qualified to determine the most eligible way of classifying them in the asylum. Some might be permitted with impunity, and even advantage, to associate with the inmates generally; others, on the contrary, it might be necessary to confine entirely to the criminal ward and airing-court connected with it. To suppose that some part of a great county asylum cannot be appropriated to such a purpose, and rendered sufficiently secure to guarantee the safe custody of any class of dangerous lunatics, appears to me unaccountable. And, after all, when thus distributed, the number of criminal lunatics which would be sent to each county asylum would be very few; and whatever may have been their offences, they ought to be treated with as much consideration as ordinary patients. Many of these cases, indeed, professionally considered, are extremely interesting to the physician engaged in this department of medical practice. Besides which, as regards their moral management, it is well known that criminal lunatics—the designation of whom as criminals excites so much apprehension—are often the most quiet, docile, and inoffensive persons in these establishments. My experience upon this point accords with that of Dr. Bucknill, who has, in his 'Inquiry,' published a series of cases, to which he 'confidently refers as proof, that the most criminally disposed lunatics are not the so-called criminal lunatics, and that the majority of the latter are as tractable and harmless as the average of insane persons to whom the stigma of crime has never been attached.'"

Dr. Hood dwells upon the importance, in a curative, as well as in a financial point of view, of compelling persons confined as criminal lunatics to occupy themselves in some kind of productive employments consonant with their former habits and stations of life, having a due regard to the capacities, physical and mental, of the patients whose strength should not, for the sake of any pecuniary advantage which the institution may derive, be overtaxed:—

"Under any circumstances, whatever system of moral management be adopted in the treatment of criminal lunatics, it should be characterised by the greatest possible amount of kindness and benevolence. They are a class, owing to the *tristesse* of their position, more sensitive, more susceptible, and more excitable than ordinary patients, and many of them endure more mental suffering. It is impossible to imagine the sad thoughts and painful associations which may, with the returning light of reason, or during a lucid interval, recur to the mind of these afflicted persons, who may be fully conscious, and bitterly lament the wrong which they may recollect having committed. Many who are partially recovered become victims of melancholia—'*Io! vaga tristis Orestes!*' It was suggested by Horace himself, that the most atrocious crimes which were invented by the genius of heathen fiction might be exculpated upon the plea of insanity.

"'An tu reris eum occisâ insannîsse parente,
Ac non antè malis dementem actum Furiis, quàm
In matris jugulo ferrum tepefecit acutum?
Quin, ex quo est, habitus malè tutæ mentis Orestes,
Nil sanè fecit quod tu reprêndere possis.'*

"The liberation of criminal lunatics upon recovery, suggests a very serious and difficult subject for consideration, inasmuch as the greatest possible precaution should be taken that the safety of the public shall not be endangered by their being prematurely discharged."

When referring to the many popular prejudices that prevail with regard to the treatment and condition of criminal lunatics, Dr. Hood alludes to the prevalent opinion, that many such patients are detained in Bethlehem Hospital, and other lunatic asylums, for long periods after they have sufficiently recovered, and are safe to be at large. Hadfield's case has been cited as an illustration of the fact. We subjoin the following interesting particulars of this criminal's case:—

"Upon inquiring into the history of Hadfield, it will be found, that although he had a perfect recollection of every incident connected with his attempt upon the life of George III., and described with much enthusiasm the zeal with which the illustrious Erskine pleaded his defence, expressing the utmost gratitude towards him for his extraordinary exertions upon that memorable occasion; yet, in the recital itself of every trifling detail, he evinced a morbid pleasure not consonant with an exactly sane state of mind. He used to relate with much vivacity and self-satisfaction an anecdote which occurred at the doors of the theatre, which he considered an excellent joke. In consequence of his Majesty's expected visit, an immense concourse of people had assembled round the pit doors of the theatre, and when they were thrown open, the crowd made a prodigious rush—as might be supposed—into the

* "Do you imagine that Orestes grew mad after the parricide, and was not distracted and haunted by execrable Furies before he warmed the pointed dagger in his mother's blood? *Nay, from the time that you supposed him out of his senses, he really did nothing that you can blame.*" This curious passage occurs, Sat. 3, Book ii., L. 134, *et seq.* Matthias Gesner subjoins the following note—"*Furor non fuit pœna parricidii sed causa.*" Edinburgh. Ed. 1806. P. 377.

house, in the midst of which a young woman, immediately behind Hadfield, cried out, 'Oh, sir! the handle of your umbrella is running into my breast.' 'But'—Hadfield was wont to add, laughing—'the handle of my umbrella was the butt end of the pistol!'

"There can be no doubt that this man was a very dangerous lunatic, his homicidal propensities having been excited by religious delusions, which were suggested to his mind by another lunatic, who was a cobbler, named Bannister Truelock. It is impossible to account for the extravagance even of insane delusions. Hadfield imagined that he was to be God, and the cobbler Truelock, Satan, and that a happy change would then take place throughout the world. This millennium, Truelock persuaded Hadfield, would be hastened by the death of the reigning Sovereign, and the deluded man forthwith furnished himself with a pistol to accomplish the projected deed. It should be remembered, however, that Hadfield, before this regicidal attempt, had betrayed the most inveterate homicidal propensities. One night he seized his own child—a boy of eight years of age—with the resolution of dashing his brains out, which he was prevented doing by the forcible interposition of the mother; and on several occasions he threatened to murder his wife. In a work entitled 'Sketches of Bedlam,' I find it stated that 'Hadfield was confined, as a maniac, in the old Bethlem, where during his stay he killed a poor maniac named Benjamin Swain, by a stroke over his head, which tumbled him over a form, and he died instantly. He contrived to make his escape from old Bethlem, but was apprehended at Dover, and for his better security was sent to Newgate, where he remained until the 8th November, 1816, when he was brought here.' As far as the apparent rationality of Hadfield was concerned, it cannot be received as any criterion of his sanity—or accepted as evidence that the homicidal propensity was at an end and would never recur; he was, from the observations reported to me, by no means considered safe by those who watched him; he was often morose and sullen, gave way to gusts of passion and sudden impulses, which, bearing in mind his previous history, rendered him, in my opinion, a very unfit subject for liberation."

In conclusion, whilst directing the particular attention of our readers to this interesting and able addition to British psychological literature, we quote a recapitulation of the author's principal suggestions in reference to the future provision of criminal lunatics:—

"*First*—That in the distribution of criminal lunatics a principle of classification should be recognised, and that the highest class of offenders should, under the Queen's warrant, be confined, either in Bethlehem or in some other recognised State asylum.

"*Second*—That criminal lunatics who have committed offences of a minor description, should be confined under the Secretary of State's warrant in the county asylums which are established in the counties to which they respectively belong.

"*Third*—That—for the reasons above assigned—it being inexpedient to erect a central lunatic asylum, every county asylum should be required to provide a special ward and airing court in connexion with it, where the safe custody of this class of patients shall be insured.

"*Fourth*—That the association of criminal lunatics with other patients must depend upon circumstances, which should be left to the discretion of the superintendent or medical officer of the asylum, whose reasons for allowing such association should in every case be submitted to the Commissioners in Lunacy for their consideration and approval.

"*Fifth*—That convicts becoming insane in prisons should not be sent to county asylums, but that a criminal ward, or some other appropriate place, in connexion with the infirmary of the prison, should be appointed for the confinement of such patients, who should be placed under the immediate charge of the medical officer of the prison, and there remain under his treatment until the term of their imprisonment has expired, a report of the case being at the same time forwarded to the Commissioners in Lunacy, to be by them transmitted to the Secretary of State.

"*Sixth*—That criminal lunatics, when received into lunatic asylums by the warrant of the Secretary of State, should be placed under the *immediate* jurisdiction of the Commissioners in Lunacy, who should be empowered to require periodical reports from the superintendents and medical officers of such asylums respecting their bodily and mental state of health. Furthermore, that the Commissioners in Lunacy should be empowered to take measures for the discharge of such criminal lunatics as recover their reason, whether by reporting their state of sanity to the Secretary of State, or otherwise; and that they should have the entire control over the classification and general management of this class of patients."

